# Performance of the 2007 WHO Algorithm to Diagnose Smear-Negative Pulmonary Tuberculosis in a HIV Prevalent Setting

**DOI:** 10.1371/journal.pone.0051336

**Published:** 2012-12-19

**Authors:** Helena Huerga, Francis Varaine, Eric Okwaro, Mathieu Bastard, Elisa Ardizzoni, Joseph Sitienei, Jeremiah Chakaya, Maryline Bonnet

**Affiliations:** 1 Clinical Research Department, Epicentre, Paris, France; 2 Medical Department, Médecins Sans Frontières, Paris, France; 3 District Hospital Laboratory, Médecins Sans Frontières, Homa Bay, Kenya; 4 Mycobacteriology Unit, Institute of Tropical Medicine, Antwerp, Belgium; 5 Division of Leprosy, Tuberculosis and Lung Disease, Ministry of Health, Nairobi, Kenya; 6 Centre for Respiratory Diseases Research, Kenya Medical Research Institute, Nairobi, Kenya; National Institute for Infectious Diseases (L. Spallanzani), Italy

## Abstract

**Background:**

The 2007 WHO algorithm for diagnosis of smear-negative pulmonary tuberculosis (PTB) including *Mycobacterium tuberculosis* (MTB) culture was evaluated in a HIV prevalent area of Kenya.

**Methods:**

PTB smear-negative adult suspects were included in a prospective diagnostic study (2009–2011). In addition, program data (2008–2009) were retrospectively analysed. At the first consultation, clinical examination, chest X-ray, and sputum culture (Thin-Layer-Agar and Lowenstein-Jensen) were performed. Patients not started on TB treatment were clinically re-assessed after antibiotic course. The algorithm performance was calculated using culture as reference standard.

**Results:**

380 patients were included prospectively and 406 analyzed retrospectively. Culture was positive for *MTB* in 17.5% (61/348) and 21.8% (72/330) of cases. Sensitivity of the clinical-radiological algorithm was 55.0% and 31.9% in the prospective study and the program data analysis, respectively. Specificity, positive and negative predictive values were 72.9%, 29.7% and 88.6% in the prospective study and 79.8%, 30.7% and 80.8% in the program data analysis. Performing culture increased the number of confirmed TB patients started on treatment by 43.3% in the prospective study and by 44.4% in the program data analysis. Median time to treatment of confirmed TB patients was 6 days in the prospective study and 27 days in the retrospective study. Inter-reader agreement for X-ray interpretation between the study clinician and a radiologist was low (Kappa coefficient = 0.11, 95%CI: 0.09–0.12). In a multivariate logistic analysis, past TB history, number of symptoms and signs at the clinical exam were independently associated with risk of overtreatment.

**Conclusion:**

The clinical-radiological algorithm is suboptimal to diagnose smear-negative PTB. Culture increases significantly the proportion of confirmed TB cases started on treatment. Better access to rapid *MTB* culture and development of new diagnostic tests is necessary.

## Introduction

Diagnosis of tuberculosis (TB) remains an important challenge to control TB particularly in resource-limited settings. Sputum smear-microscopy has a low sensitivity especially in Human Immunodeficiency Virus (HIV) infected patients (24%–61%) [1]–[5]. However, this is often the only test available to confirm pulmonary TB (PTB) in these settings. Clinical and radiographic criteria have a limited performance to diagnose TB in HIV infected patients [6], [7]. Sputum *Mycobacterium tuberculosis* (MTB) culture is the gold standard for smear-negative TB diagnosis, but requires a good level of infrastructure and technical expertise. Moreover, conventional culture methods such as Lowenstein-Jensen (LJ) are slow and have limited impact on therapeutic decision. Modern culture methods on liquid media such as Mycobacteria Growth Indicator Tube (MGIT), are more sensitive and faster [8]–[10], but have more risk of contamination compared to LJ [11], [12]. Alternative non-commercial rapid culture methods, such as Microscopic Observation Drug Susceptibility (MODS) or Thin Layer Agar (TLA), have recovery rates comparable to other techniques [13]–[18], and might be more suitable to resource-limited settings. Recently, a closed automated polymerase chain reaction (PCR) test, Xpert MTB/RIF assay, has shown good detection rates with the potential to be used at district health facility level [19].

In 2007, WHO revised the smear-negative PTB diagnostic algorithm in order to improve PTB diagnosis in HIV prevalent and resource-constraint settings [20]. The revised guideline recommended performing a chest X-ray before the antibiotic trial course and using rapid culture techniques when feasible. The revised algorithm was implemented at the Homa Bay District Hospital, a HIV prevalent area in Western Kenya. Médecins Sans Frontières (MSF) in collaboration with the supranational reference laboratory of the Institute of Tropical Medicine Institute in Antwerp (Belgium) and the Ministry of Health of Kenya set up a MTB culture laboratory introducing TLA and LJ methods. Recently, WHO have recommended using Xpert MTB/RIF as the initial diagnostic test for TB [21]. However, this new technology is barely available and clinical-radiological algorithms are largely used to diagnose smear-negative PTB in the peripheral sites of resource-limited settings.

Few studies have assessed the performance of the 2007 WHO revised diagnostic algorithm in HIV prevalent settings [22]–[25]. We conducted a field evaluation of the algorithm in outpatient smear-negative PTB suspects in Homa Bay to assess the performance of the clinical-radiological algorithm for the initiation of TB treatment using culture as reference standard and to compare the proportion of confirmed TB patients started on TB treatment using the diagnostic algorithm with and without culture.

## Methods

### Study design

A prospective diagnostic study was conducted. In addition program data were retrospectively collected from a period before the start of the prospective study.

### Prospective study

#### Setting and population

The Homa Bay District Hospital is the reference health facility for a district of 350,000 people. TB and HIV care are supported by MSF. Between September 2009 and February 2011 all consecutive smear-negative PTB suspects attending the hospital TB clinic were eligible to the study if they were 15 years or more of age, presented with a cough of at least 2 weeks, had 2 negative sputum smears without any positive, and lived within the hospital TB clinic catchment area for TB treatment (10 km radius). Patients who had taken fluoroquinolones or anti-TB drugs in the last one month were excluded.

#### Diagnostic algorithm

**Figure 1:**
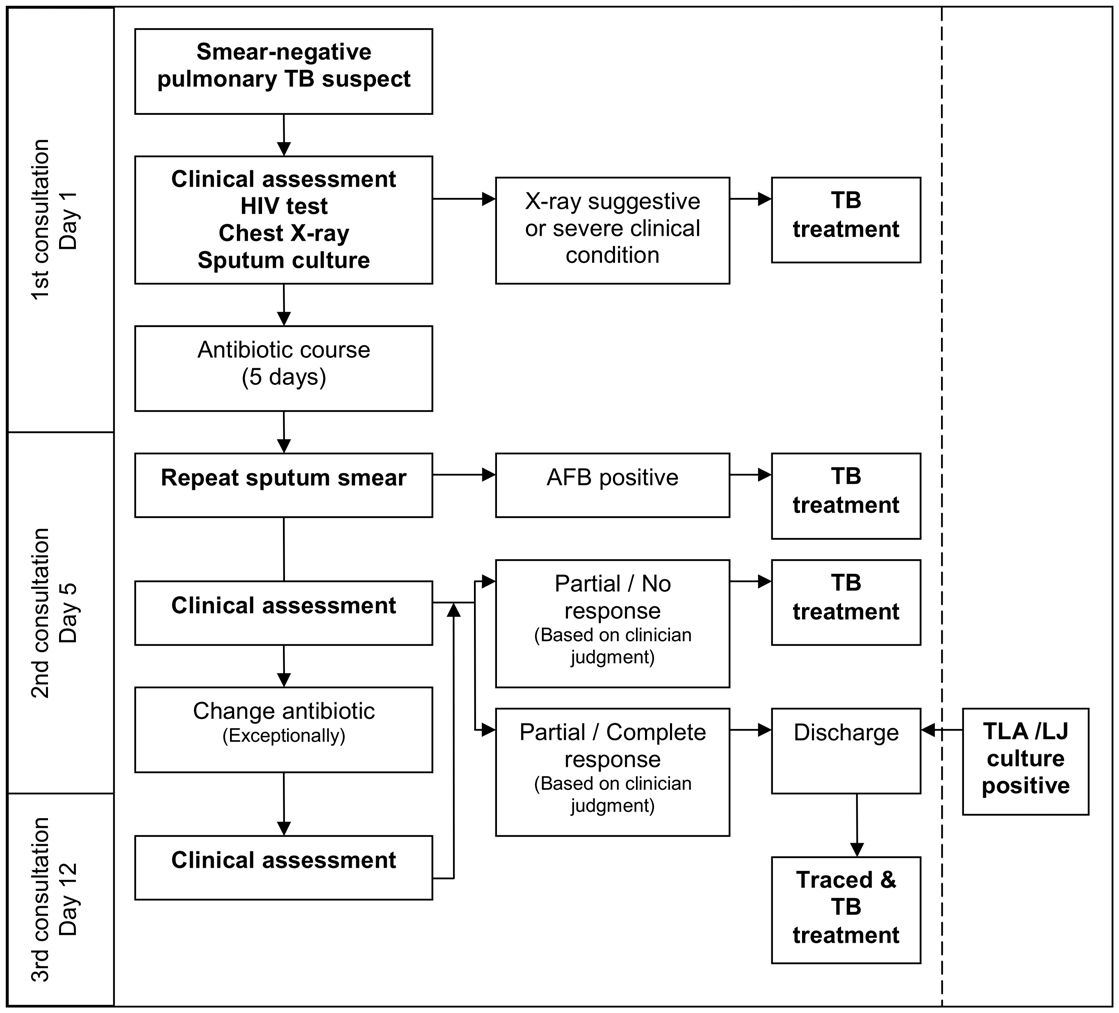
Diagnostic algorithm for smear-negative TB suspects (prospective study).

Three sputum samples collected one on spot and two early in the morning the following day, were examined with smear microscopy in all PTB suspects. Smear-positive patients were started on TB treatment and not included in the study. Culture was systematically performed for smear-negative patients. At the 1^st^ consultation, all smear-negative patients were clinically assessed by a trained clinical officer, and a chest X-ray was performed (figure 1). Patients with a chest X-ray suggestive of TB or presenting highly suggestive TB symptoms and in severe clinical condition were started on TB treatment. Patients not fulfilling these conditions were given an antibiotic (amoxicillin 1 g 3×/day 5 days). HIV testing with pre and post test counselling was offered to all TB suspects. At the 2^nd^ consultation, five days later, patients were re-assessed clinically, sputum smear microscopy examination was repeated and a clinical decision to or not to start TB treatment made. Exceptionally, a second antibiotic was prescribed for one week followed by a 3^rd^ clinical consultation. At anytime, patients with positive culture not already on treatment were contacted or traced at home to start TB treatment as soon as possible. Patients who missed study appointments were actively traced. Patients started on TB treatment received 2 months of isoniazid, rifampicin, ethambutol and pyrazinamide (HREZ) and 4 months HR according to the Ministry of Health guidelines [26]. Treatment was delivered under self administrative therapy combined with patient-centred adherence strategies, with good treatment adherence as previously reported [27]. All tests and treatment were free of charge.

#### Study procedures

In order to assess clinical response after the antibiotic course, the clinician used at each consultation a score grading the patients' symptoms severity between 1 (mild) and 4 (life-threatening). Complete resolution was defined as the resolution of all clinical symptoms with a normal physical examination; partial resolution as a clinical improvement with persistence of clinical symptoms/signs; and no resolution as the absence of improvement or clinical worsening. Severe clinical condition, irrespectively of TB diagnosis, was defined by one of the following signs: respiratory rate >30/minute, temperature >39°C, tachycardia >120/minute, or unable to walk without help. Malnutrition was defined according to the body mass index (BMI): BMI <18.5 and >17: mild; BMI ≤17 and >16: moderate; BMI ≤16: severe.

Chest X-rays were interpreted by the study clinician. A second reading by an external radiologist blind to the patient's clinical data and to the clinician's interpretation was performed at the end of the study to measure the inter-reader agreement. A tick sheet including pictograms was used for reporting X-ray results by both the clinician and the radiologist (Appendix). Results were defined as “Suggestive of TB” by the presence of upper lobe infiltrate, cavitation, hilar adenopathy, pleural effusion or miliary pattern; “Compatible with TB” when any other sign that the clinician judged was compatible with TB was present and “Not TB” if the X-ray was normal or if the clinician judged that the signs present were not suggestive/compatible with TB [20].

Light Emitting Diode-based fluorescence microscopy after auramine [28] staining was performed. At least 1 AFB/100HPF was considered as positive. In case of negative results, each of the two best quality sputum samples was cultured on both TLA and LJ media. Prior inoculation specimens were decontaminated using NALC-sodium hydroxide method with 1% final concentration. Para-nitrobenzoic acid added to TLA during medium preparation was used to distinguish between MTB and non-TB mycobacterium (NTM). Identification of colonies from LJ was performed by subculturing on a TLA plate. After inoculation TLA plates were incubated in a 5% CO2 incubator and LJ in a standard incubator at 37°C. TLA plates were read twice a week for six weeks using a standard light microscope and LJ tubes once a week for eight weeks by trained laboratory technicians. Quality control of positive cultures was done at the Central Reference Laboratory in Nairobi and the supranational TB laboratory at the Institute of Tropical Medicine in Antwerp, Belgium. Growth of at least 1 MTB microcolony defined a positive TLA result and 1MTB colony a positive LJ result.

A patient was defined as culture positive if at least one of the four culture results (LJ or TLA) was positive and culture negative if at least 2 culture results were negative and none was positive. NTM result was considered as MTB culture negative result. Patients with culture contaminated results (≥3 contaminated without any positive results) were excluded from this analysis. Patients with positive culture were considered as confirmed TB cases.

Determine® HIV Rapid Test and Uni-Gold® HIV Rapid Test were used in succession for HIV testing and Facscount machine for CD4 count.

### Retrospective programmatic assessment

Program data accrued between January 2008 and August 2009 were collected retrospectively from patients' files and clinic registers. Data included socio-demographic information, treatment decision by the clinician and culture results. Compared to the prospective study, the program clinical officer did not receive specific training on chest-ray reading, pictogram tick sheets were not used for chest-ray interpretation, antibiotic trial was not standard, there was no grading of the severity of symptoms, and no standard case definition of clinical response to the antibiotic course. Microscopy and MTB culture were done in similar conditions. MTB culture positive patients not on TB treatment were traced to start treatment. There was no tracing for patients missing clinic appointments. Chest-rays were not free of charge.

### Sample size and statistical analysis

Sample size estimates was based on the comparison of the proportion of patients appropriately started on TB treatment (MTB culture confirmed) using the diagnostic algorithm with and without culture in the same population of PTB suspects. This was possible since culture results were expected after the therapeutic decision by the clinician had been made using clinical and radiological findings. Using a Mc Nemar's one side test of equality of paired proportions (nQuery Advisor® 5.0) with an estimation of a minimum increase of 5% of patients adequately started on treatment using the algorithm with culture and 10% proportion of total discordant pairs, a risk alpha of 0.05 and a risk beta of 0.20, a minimum of 231 patients were required. The sample size was increased to 380 patients to stratify the analysis in HIV infected patients (70%) and take into account dropouts (15%). All consecutive PTB suspects attending the clinic during the same duration of time than the one used for the prospective study were included in the retrospective analysis.

Data were double entered using Epi-Data 3.0 software (The EpiData Association, Odense Denmark) and analyzed in Stata® 10.0 software (College Station, Texas, USA).

The proportion of confirmed TB patients started on TB treatment using the 2007 WHO algorithm with and without considering culture results was calculated. The performance (sensitivity, specificity, predictive values and likelihood ratios) of the diagnostic algorithm without culture was calculated using positive culture as reference standard. Receiver operating characteristic (ROC) analyses were performed and the area under the curve (AUC) calculated. Results were compared between prospective research and routine program conditions. Multivariate logistic regressions were performed to identify factors associated with overtreatment (culture negative patients started on TB treatment) and undertreatment (culture positive patients not started on TB treatment) when using the diagnostic algorithm without culture. Inter-agreement of chest X-ray reading was assessed by measuring the kappa coefficient and interpreted according to Landis and Koch classification [29]. Chi-2 test and Wilcoxon tests were used to compare qualitative and quantitative and qualitative data between independent populations. Since the diagnostic algorithms with and without MTB culture were assessed in the same patients, we used the McNemar test to compare the proportions of confirmed TB patients started on TB treatment using the diagnostic algorithm with and without culture. Results were presented with a 95% confidence interval (95% CI).

#### Ethics statement

The protocol for both the prospective study and the retrospective program data analysis was approved by the Kenya Medical Research Institute Ethical Review Committee and by the Ethical Review Committee at the “Comité de Protection des Personnes”, Saint Germain en Laye, in France”. Written informed consent was obtained from all study participants as well as from guardians on the behalf of the minor participants before enrolment in the prospective study. The written informed consent was approved by the Ethics Committees. No consent was sought for patients included in the retrospective program data analysis.

## Results

### Prospective study

#### Patient's characteristics

During the prospective study period, 583 smear-negative PTB suspects were consecutively screened, 380 (65%) fulfilled the inclusion/exclusion criteria and were included in the study. Patients' characteristics, clinical and chest X-ray findings are presented in table 1. Overall, 68.0% of the 363 TB suspects with a known HIV status were HIV positive. Of 238 patients with a WHO staging available, 74.4% were in stage 3 or 4. The median CD4 count (N = 225 patients) was 274 cells/µl (IQR: 145–501) and 83 (35.4%) had less than 200 cells/µl. HIV positive patients were more often malnourished or in severe clinical condition, and had more pathological signs at the physical examination than HIV negative patients.

**Table 1:** Patients' characteristics of the smear-negative PTB suspects included in the prospective study.

	*All patients (N = 380)*	*HIV positive (N = 247)*	*HIV negative (N = 116)*	*p*
Age, median years (IQR)	34 (26–48)	34 (28–46)	37.5 (22–60)	0.469
Female, n (%)	239 (62.9)	159 (64.4)	71 (61.2)	0.559
History of TB, n (%)	92 (24.2)	70 (28.3)	21 (18.1)	0.036
Antibiotic in the past 2 weeks, n (%)	92 (24.2)	59 (23.9)	29 (25.0)	0.611
Amoxicillin	42 (45.6)			
Erythromycin	20 (21.7)			
Cotrimoxazole	21 (22.8)			
Other	9 (59.8)			
BMI, median^a^, (IQR)	19.7 (18.3–21.5)	19.3 (17.9–20.9)	20.5 (19.0–22.5)	**<0.001**
Malnourished, n^b^ (%)	107 (28.2)	86 (34.8)	20 (17.2)	**<0.001**
Mild malnutrition	64 (59.8)	49 (57.0)	15 (75.0)	
Moderate malnutrition	20 (18.7)	17 (19.8)	3 (15.0)	
Severe malnutrition	23 (21.5)	20 (23.3)	2 (10.0)	
Severe clinical condition, n (%)	27 (7.1)	23 (9.3)	3 (2.6)	**0.021**
No. of symptoms, median (IQR)	7 (6–8)	7 (6–8)	6 (5–8)	0.085
No. signs at physical exam, median (IQR)	3 (2–4)	3 (3–4)	2 (2–3)	**<0.001**
Symptoms, n (%)				
Fever	331 (87.1)	220 (89.1)	97 (83.6)	0.146
Weight loss	309 (81.3)	213 (86.2)	82 (70.7)	**<0.001**
Night sweats	299 (78.7)	203 (82.2)	85 (73.3)	**0.050**
Loss of appetite	245 (64.5)	175 (70.9)	61 (52.6)	**<0.001**
Haemoptysis	64 (16.8)	41 (16.6)	19 (16.4)	0.958
Clinical examination, n (%)				
Adenopathies	238 (62.6)	195 (79.0)	35 (30.2)	**<0.001**
Temperature ≥37.5°C	42 (11.1)	35 (14.2)	5 (4.3)	**0.005**
Ascitis	5 (1.3)	2 (0.8)	2 (1.7)	0.463
X-ray findings, n (%)				
Infiltration	284 (74.7)	191 (77.3)	81 (69.8)	0.124
Hilar adenopathy	196 (51.6)	141 (57.1)	49 (42.2)	**0.008**
Consolidation	87 (22.9)	67 (27.1)	17 (14.7)	**0.009**
Cavities	29 (7.6)	20 (8.1)	8 (6.9)	0.689
Pleural effusion	24 (6.3)	18 (7.3)	5 (4.3)	0.278
Miliary pattern	5 (1.3)	4 (1.6)	0 (0.0)	0.168
Apical fibrocystic	2 (0.5)	2 (0.8)	0 (0.0)	0.331

a . BMI: body mass index.

b . Malnourished: BMI<18.5.

Thirty two patients did not have final culture result because cultures were not performed (n = 14) or were contaminated (n = 18). Out of the remaining 348 patients, 61 (17.5%) were positive, 276 (79.3%) negative and 11 (3.2%) had NTM results (Table 2). Out of 233 HIV infected TB suspects, 48 (20.6%) were culture positive. The median delay from inoculation to a positive result was 15 days (IQR: 11–21) with TLA and 23 days (IQR: 17.5–31.5) with LJ.

**Table 2:** Culture results of smear-negative PTB suspects, prospective study.

	*LJ*				
*TLA*	Positive	Negative	NTM	Contaminated	Total^a^
Positive	34	16	0	1	51
Negative	10	256	6	17	289
NTM	0	4	1	0	5
Contaminated	0	14	0	5	19
Total	44	292	7	23	364

a . In 2 patients LJ was negative and TLA was not performed.

#### Diagnostic and treatment pathway

**Figure 2:**
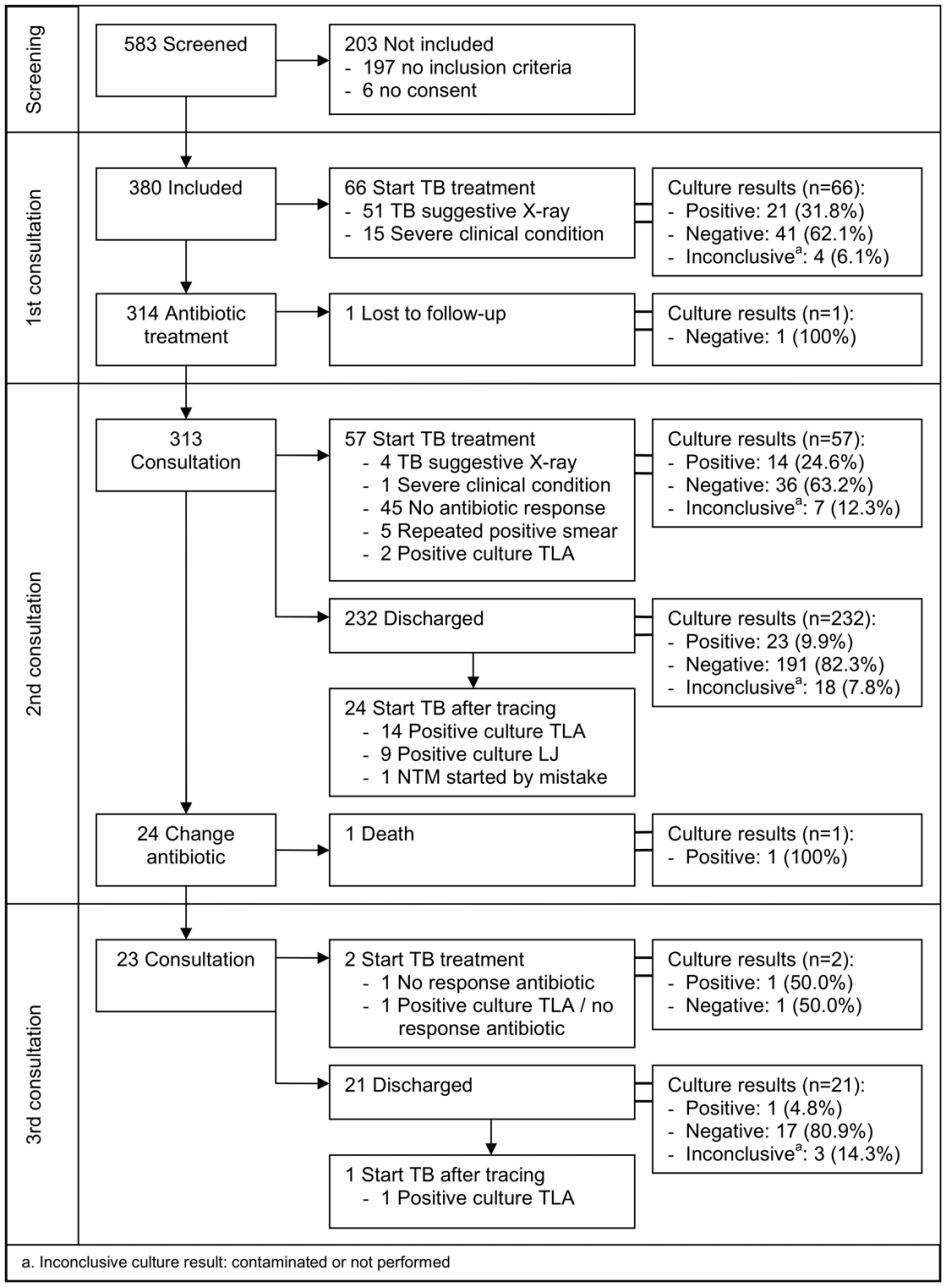
Diagnostic and treatment pathway for smear-negative TB suspects (prospective study, N = 380).

Figure 2 presents the diagnostic and therapeutic pathway of the prospective study. In total, 150 (39.5%) PTB suspects were started on TB treatment. Of the 139 PTB suspects with a final culture result started on TB treatment, 60 (43.2%) were confirmed TB cases.

The reasons and median time to start TB treatment among culture positive smear-negative PTB patients are shown in Table 3. The median time to start confirmed TB patients on treatment was 6 days (IQR: 0–27).

**Table 3:** Reasons to start treatment among smear-negative culture confirmed PTB cases in the prospective study and in the retrospective analysis of program data.

	*Prospective study (N = 60)* a		*Retrospective program data (N = 72)*	
	*N (%)*	*Median time to treat (days)*	*N (%)*	*Median time to treat (days)*
Started on TB treatment due to clinical signs and/or X-ray:	33 (55.0)	0	23 (31.9)	1
Chest X-ray suggestive	19 (31.7)	0		
Severe clinical condition	3 (5.0)	0		
Repeated smear positive	2 (3.3)	6		
No response to antibiotic	9 (15.0)	6		
Started on TB treatment due to culture result:	26 (43.3)	31	32 (44.4)	34.5
Culture TLA positive	17 (28.3)	22	20 (27.7)	30.5
Culture LJ positive	9 (15.0)	39	12 (16.7)	50
Not started on TB treatment	1 (1.7)	-	17^b^ (23.6)	-

a . In one patient, the reason could not be discerned (TLA result available the day of the third consultation).

b . 4 had died by the time they were traced, 2 did not come to the clinic to start treatment, 1 refused treatment, 3 were not found during the tracing and for 7 there was no information about the tracing.

The proportion of confirmed TB cases started on treatment increased from 55.0% using the clinical algorithm without culture to 98.3% using the algorithm with culture (p<0.001). Including culture in the algorithm increased the proportion of confirmed TB cases started on treatment by 43.3% (95%CI: 29.1–57.5). In HIV positive patients including culture increased the proportion of treated cases by 38.3% (95%CI: 22.3–54.3).

#### X-ray interpretation and antibiotic course

The study clinician interpreted 59 (15.5%) X-rays as “suggestive of TB”, 196 (51.6%) as “Compatible with TB”, and 125 (32.9%) as “Not TB”. Patients with an X-ray interpreted as “Suggestive of TB” were more likely to be MTB culture confirmed (OR: 3.4, 95%CI: 1.8–6.4, p<0.001) than the ones with X-ray interpreted as “Compatible with TB” or “Not TB”. Seven percent of the patients with a chest X-ray interpreted as “Not TB” were culture positive. The inter-reader agreement for X-ray interpretation between the clinician and radiologist was low (Kappa coefficient = 0.11, 95%CI: 0.09–0.12). X-rays were interpreted as “Not TB” in 73% of the cases by the radiologist and in 33% of the cases by the clinician. X-ray interpretation as “suggestive of TB” by the radiologist had a higher specificity compared to the same interpretation by the study clinician (98.5% versus 87.0%, p<0.001).

Of the 313 PTB suspects who received an antibiotic, 85%, had a partial response. Patients with no response to antibiotic were more likely to be culture confirmed (OR: 3.8, 95%CI: 1.3–10.8, p = 0.014) than patients with a complete or partial response. However, 9% of the culture confirmed PTB patients who received an antibiotic had a complete response.

### Retrospective analysis

A total of 406 smear negative PTB suspect patients were included in the retrospective analysis: 213 (52.5%) females, median age 32 years (IQR: 25–45). Of the 98 with an HIV test result available, 71 (72.5%) were positive. A chest X-ray was performed in 94 (23.2%) of the patients. Out of the 330 patients with a final culture result, 72 (21.8%) were positive, 257 (77.9%) negative, and 1 (0.3%) had an NTM result. A total of 125/406 (30.8%) patients were started on treatment and 55 (44%) had a confirmed TB.

The reasons and median time to start TB treatment among culture positive smear-negative PTB patients are shown in Table 3. The median time to start confirmed TB patients on treatment was 27 days (IQR: 3–35).

The proportion of confirmed TB cases started on treatment increased from 31.9% using the clinical algorithm without culture to 76.4% using the algorithm with culture (p<0.001). Including culture in the algorithm increased the proportion of confirmed TB cases started on treatment by 44.4% (95%CI: 31.6–57.3). The proportion of culture confirmed patients not started on treatment was 23.6% in program conditions (retrospective analysis) versus 1.7% in the prospective study, p<0.001.

#### Algorithm performance

**Figure 3:**
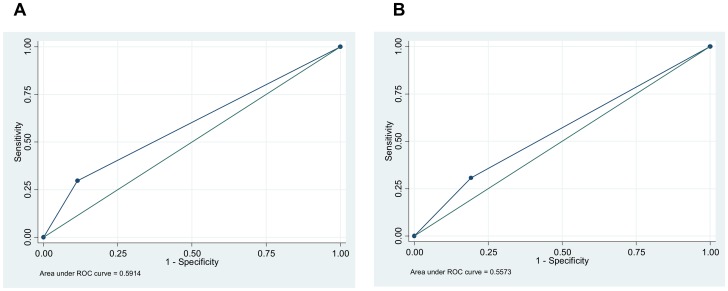
Clinical-radiological algorithm ROC curves. ROC curve for the clinical-radiological smear-negative diagnostic algorithm without culture in the prospective study (A) and in the retrospective analysis of program data (B).

The performances of the clinical-radiological diagnostic algorithms in the prospective study and the retrospective analysis are presented in Table 4. The sensitivity was significantly lower in program conditions, 31.9% compared to 55.0% in the prospective analysis (p = 0.008). The positive predictive value was similar in both, 30.7% and 29.7% respectively. The negative predictive value was significantly lower in program conditions (p = 0.02). The positive and negative likelihood ratio was 1.6 (95%CI: 1.1–2.4) and 0.9 (95%CI: 0.7–1.0) in program conditions and 2.0 (95%CI: 1.5–2.7) and 0.6 (95%CI: 0.5–0.8) in the prospective analysis. The AUC for this algorithm was 0.59 (95%CI: 0.53–0.64) in the prospective study and 0.56 (95%CI: 0.50–0.61) in the retrospective analysis (Figure 3). Using data from the prospective study, past TB history, number of symptoms and signs at the clinical exam were independently associated with risk of overtreatment (Table 5). No factor was found associated with undertreatment.

**Table 4:** Performance of the 2007 WHO diagnostic algorithm for smear-negative PTB without culture in the prospective study and the retrospective analysis of program data (reference: positive culture).

	*Culture positive*	*Culture negative*	*Sensitivity, % (95% CI)*	*Specificity, %*	*Positive predictive value, %*	*Negative predictive value, %*
**Prospective study (N = 347)** a			55.0 (41.6–67.9)	72.8 (67.3–77.9)	29.7 (21.4–39.1)	88.6 (83.8–92.3)
TB start	33	78				
Not start	27	209				
**Retrospective program data (N = 330)**			31.9 (21.4–44.0)	79.8 (74.4–84.6)	30.7 (20.5–42.4)	80.8 (75.4–85.4)
TB start	23	52				
Not start	49	206				

a . One patient excluded since reason to start TB treatment could not be discerned.

**Table 5:** Factors associated with overtreatment when the diagnostic algorithm without culture was used (prospective study, N = 303).

	*Univariate analysis*			*Multivariate analysis*		
	OR	95% CI	p	OR	95% CI	p
Age (/year)	1.01	0.99–1.02	0.326			
Gender (female vs male)	0.65	0.39–1.10	0.108			
History of TB (yes/no)	3.21	1.85–5.55	**<0.001**	3.42	1.84–6.35	**<0.001**
Antibiotics in last 2 weeks (yes/no)	0.79	0.43–1.46	0.453			
BMI (/1 unit)	0.92	0.84–1.00	**0.041**			
No. symptoms reported (/1 symptom)	1.83	1.47–2.29	**<0.001**	1.87	1.47–2.37	**<0.001**
No. signs at clinical exam (/1 sign)	2.01	1.42–3.09	**<0.001**	1.77	1.16–2.71	**0.008**
Severe condition (yes/no)	5.99	2.58–13.84	**<0.001**			
HIV(positive/negative)	2.24	1.18–4.25	**0.014**			

## Discussion

In this study, the proportion of smear-negative confirmed PTB patients started on treatment using the 2007 WHO clinical-radiological algorithm without culture was low: 55% in the optimised prospective study and 32% in the retrospective analysis of program data. The clinical-radiological algorithm had poor discrimination capacity as shown by the low AUC in the ROC analyses. In Homa Bay, the clinical-radiological algorithm led to a high proportion of TB cases missed and a high proportion of patients overtreated. Our study is in line with most of the studies carried out in similar settings [6], [22], [25], [30] which have shown low algorithm sensitivity ranging from 23% to 59%. However, sensitivities of 80–95% have also been reported [23], [24].

Less than one third of patients treated for TB were confirmed with TB in research and routine conditions. The use of rapid culture did not reduce the risk of overtreatment since majority of patients were started on treatment before the culture results. Indeed, the time to positive results for rapid culture (15 days) might be still too long to have an impact on the risk of overtreatment. Other studies [6], [22], [24], [25] have shown similar low positive predictive values (22%–52%). Only one study has shown a much higher positive predictive value of 90% [23]. As empiric treatment in patients with no response to antibiotic trial and negative investigations is part of the WHO algorithm, overtreatment is likely. In addition, the fear of missing TB, especially in HIV infected patients along with the difficulty to diagnose TB using only clinical-radiological signs may contribute to overtreatment. Patients having a history of TB and polysymptomatic patients were at higher risk of been wrongly started on TB treatment by the study clinician even when these factors are not predictors of TB [6], [31], [32].

When MTB culture was included in the algorithm, the number of patients correctly started on TB treatment increased by 43–44% in the prospective study and the retrospective analysis. During the prospective study all culture confirmed TB cases but one were started on TB treatment, while in routine conditions almost one forth of the cases were not started on treatment despite having a positive culture result. This was probably the result of a less effective tracing in program conditions. In addition, the delay to start treatment among confirmed TB cases remained acceptable (6 days) in the prospective study while it was very long in routine conditions (27days) most likely due to the same reason. Similar findings have been reported in other routine settings [6], [22], [33]: in Cambodia 29% of the patients were not started on treatment despite the culture results and in Soudan enrolment into the culture programme was suspended due to the number of lost to follow-up cases. Implementing effective tracing is therefore essential when introducing MTB culture in the diagnostic algorithm.

One third of the culture positive TB cases were diagnosed through chest X-ray at the first consultation. Other studies have shown that performing the chest X-ray before the antibiotic course can reduce considerably the time to treatment and as a result potentially improve the survival of smear-negative HIV infected patients with advance disease [1], [5], [32], [34], [35]. Efforts should be done in order to have chest X-rays available and free-of-charge in the peripheral areas of resource limited-settings. Nevertheless, the performance of the chest X-ray to diagnose TB varies depending on the reader. In this study the field clinician interpretation was very different compared to the radiologist one. As described in other sites [22], [34], [35], the study clinician interpreted X-rays as pathological more often than the radiologist leading to a lower specificity to diagnose TB and a low chest X-ray reading agreement.

The diagnostic value of the response to antibiotic course is questionable [34] and its accuracy has shown diverse degrees of limitation [2], [6], [31], [36], [37]. Although complete absence of response to antibiotic was associated with TB in our study, this finding was not very helpful for decision-making since majority of the TB patients had a partial response to antibiotics.

Culture techniques used in Homa Bay laboratory reached similar or higher detection rates to those found in other settings [13]–[15]. A considerable number of patients were positive with only one of the two techniques and the implementation of both would be advisable to help in the diagnosis of pulmonary smear-negative TB.

There were some limitations to this study. The absence of an independent reference standard prevented comparing the performance of the algorithm without culture to the performance of the algorithm with culture. Although MTB culture is the only reference standard for PTB diagnosis, it remains an imperfect gold standard in smear-negative suspects, especially in HIV infected patients who are likely to have poor quality sputum specimen.

This study provides evidence of the limitations of the 2007 WHO algorithm when used without culture to diagnose smear-negative PTB with almost half of confirmed TB cases missed. The use of rapid culture is justified in HIV prevalent and resource-constraint settings as recommended by WHO [38]. Nevertheless, introducing and maintaining good culture facilities in safe conditions is a challenge for resource-limited countries [11], [12]. Feasibility and cost-effectiveness studies are lacking. The new automated PCR methods such as the Xpert MTB/RIF assay, recently endorsed by WHO for diagnosis of TB in HIV infected TB suspects is challenging the role of culture for TB diagnosis in resource-limited countries [19], [38]. However, impact and feasibility studies of diagnostic algorithms using the new PCR assays are also required.

In conclusion, the clinical-radiological diagnostic algorithm is largely suboptimal to diagnose smear-negative PTB. Culture increases significantly the proportion of confirmed TB cases started on treatment. Better access to rapid MTB culture and development of new diagnostic tests is necessary.

## Supporting Information

Figure S1 **Form used for X-ray interpretation in the prospective study.**
(PDF)

## References

[1] Getahun H, Harrington M, O'Brien R, Nunn P (2007) Diagnosis of smear-negative pulmonary tuberculosis in people with HIV infection or AIDS in resource-constrained settings: informing urgent policy changes. Lancet 369: 2042–9. 17574096 10.1016/S0140-6736(07)60284-0

[2] Cain KP, McCarthy KD, Heilig CM, Monkongdee P, Tasaneeyapan T, et al (2010) An algorithm for tuberculosis screening and diagnosis in people with HIV. N Engl J Med 362: 707–16. 20181972 10.1056/NEJMoa0907488

[3] Cattamanchi A, J. Davis L, Worodria W, den Boon S, Yoo S, et al (2009) Sensitivity and specificity of fluorescence microscopy for diagnosing pulmonary tuberculosis in a high HIV prevalence setting. Int J Tuberc Lung Dis 2009 13: 1130–6. PMC2754584 19723403

[4] Kivihya-Ndugga LE, van Cleeff MR, Githui WA, Nganga LW, Kibuga DK, et al (2003) A comprehensive comparison of Ziehl-Neelsen and fluorescence microscopy for the diagnosis of tuberculosis in a resource-poor urban setting. Int J Tuberc Lung Dis 7: 1163–71. 14677891

[5] Colebunders R, Bastian I (2000) A review of the diagnosis and treatment of smear-negative pulmonary tuberculosis. Int J Tuberc Lung Dis 4: 97–107. 10694086

[6] Davis JL, Worodria W, Kisembo H, Metcalfe JZ, Cattamanchi A, et al (2010) Clinical and radiographic factors do not accurately diagnose smear-negative tuberculosis in HIV-infected inpatients in Uganda: a cross-sectional study. PLoS One 5: e9859. 20361038 10.1371/journal.pone.0009859 PMC2845634

[7] van Cleef MR, Kivihya-Ndugga LE, Meme H, Odhiambo JA, Klatser PR (2005) The role and performance of chest X-ray for the diagnosis of tuberculosis: A cost-effectiveness analysis in Nairobi, Kenya. BMC Infectious Diseases 5: 111. 16343340 10.1186/1471-2334-5-111 PMC1326228

[8] Pardini M, Varaine F, Bonnet M, Orefici G, Oggioni MR, et al (2007) Usefulness of the BACTEC MGIT 960 System for isolation of Mycobacterium tuberculosis from sputa subjected to long-term storage. J Clin Microb 45: 575–6. 10.1128/JCM.01985-06 PMC1829068 17122020

[9] Rishi S, Sinha P, Malhotra B, Pal N (2007) A comparative study for the detection of Mycobacteria by BACTEC MGIT 960, Lowenstein Jensen media and direct AFB smear examination. Indian J Med Microbiol 25: 383–6. 18087090 10.4103/0255-0857.37344

[10] Soto A, Agapito J, Acuña-Villaorduña C, Solari L, Samalvides F, et al (2009) Evaluation of the performance of two liquid-phase culture media for the diagnosis of pulmonary tuberculosis in a national hospital in Lima, Peru. Int J Infect Dis 13: 40–5. 18555721 10.1016/j.ijid.2008.03.023

[11] Chihota VN, Grant AD, Fielding K, Ndibongo B, van Zyl A, et al (2010) Liquid vs. solid culture for tuberculosis: performance and cost in a resource-constrained setting. Int J Tuberc Lung Dis 14: 1024–1031. 20626948

[12] Mueller DH, Mwenge L, Muyoyeta M, Muvwimi MW, Tembwe R, et al (2008) Costs and cost-effectiveness of tuberculosis cultures using solid and liquid media in a developing country. Int J Tuberc Lung Dis 12: 1196–202. 18812051

[13] Robledo JA, Mejía GI, Morcillo N, Chacón L, Camacho M, et al (2006) Evaluation of a rapid culture method for tuberculosis diagnosis: a Latin American multi-center study. Int J Tuberc Lung Dis 10: 613–9. 16776447

[14] Martin A, Munga Waweru P, Babu Okatch F, Amondi Ouma N, Bonte L, et al (2009) Implementation of the thin layer agar method for diagnosis of smear-negative pulmonary tuberculosis in a setting with a high prevalence of human immunodeficiency virus infection in Homa Bay, Kenya. J Clin Microbiol 47: 2632–4. 19494065 10.1128/JCM.00264-09 PMC2725698

[15] Mejia GI, Castrillon L, Trujillo H, Robledo JA (1999) Microcolony detection in 7H11 thin layer culture is an alternative for rapid diagnosis of Mycobacterium tuberculosis infection. Int J Tuberc Lung Dis 3: 138–42. 10091879

[16] Mejía GI, Guzmán A, Agudelo CA, Trujillo H, Robledo J (2004) Cinco años de experiencia con el agar de capa delgada para el diagnostico rápido de tuberculosis. Biomedica 24 Supp 1: 52–9. 15495571

[17] Satti L, Ikram A, Abbasi S, Malik N, Mirza IA, et al (2010) Evaluation of thin-layer agar 7H11 for the isolation of Mycobacterium tuberculosis complex. Int J Tuberc Lung Dis 14: 1354–6. 20843431

[18] Martin A, Fissette K, Varaine F, Portaels F, Palomino JC (2009) Thin layer agar compared to BACTEC MGIT 960 for early detection of Mycobacterium tuberculosis. J Microbiol Methods 78: 107–8. 19427881 10.1016/j.mimet.2009.05.001

[19] Boehme CC, Nabeta P, Hillemann D, Nicol MP, Shenai S, et al (2010) Rapid molecular detection of tuberculosis and rifampin resistance. N Engl J Med 363: 1005–15. 20825313 10.1056/NEJMoa0907847 PMC2947799

[20] World Health Organisation (2007) Improving the diagnosis and treatment of smear-negative pulmonary and extrapulmonary tuberculosis among adults and adolescents: Recommendations for HIV-prevalent and resource-constrained settings. Geneva: WHO press. 44p. Available: http://whqlibdoc.who.int/hq/2007/WHO_HTM_TB_2007.379_eng.pdf. Accessed 7 June 2011.

[21] World Health Organisation (2012) Tuberculosis diagnostic Xpert MTB/RIF test. Geneva: WHO press. Available: http://www.who.int/tb/features_archive/factsheet_xpert_may2011update.pdf. Accessed 17 July 2012.

[22] Koole O, Thai S, Khun KE, Pe R, van Griensven J, et al (2001) Evaluation of the 2007 WHO guideline to improve the diagnosis of tuberculosis in ambulatory HIV-positive adults. PLoS One 6: e18502. 10.1371/journal.pone.0018502 PMC3071837 21494694

[23] Walley J, Kunutsor S, Evans M, Thoulass J, Katabira E, et al (2011) Validation in Uganda of the new WHO diagnostic algorithm for smear-negative pulmonary tuberculosis in HIV prevalent settings. J Acquir Immune Defic Syndr 57 5: e93–100. 21637111 10.1097/QAI.0b013e3182243a8c

[24] Wilson D, Mbhele L, Badri M, Morroni C, Nachega J, et al (2011) Evaluation of the World Health Organization algorithm for the diagnosis of HIV-associated sputum smear-negative tuberculosis. Int J Tuberc Lung Dis 15 7: 919–24. 21682965 10.5588/ijtld.10.0440

[25] Swai HF, Mugusi FM, Mbwambo JK (2011) Sputum smear negative pulmonary tuberculosis: sensitivity and specificity of diagnostic algorithm. BMC Res Notes 4: 475. 22044882 10.1186/1756-0500-4-475 PMC3216301

[26] Ministry of Public Health and Sanitation (2009) Guidelines on management of Leprosy and Tuberculosis. Nairobi Available: http://www.nltp.co.ke/docs/DLTLD_Treatment_Guidelines.pdf. Accessed 27 September 2012.

[27] Nackers F, Huerga H, Espié E, Aloo AO, Bastard M, et al (2012) Adherence to self-administered tuberculosis treatment in a high HIV-prevalence setting: a cross-sectional survey in Homa Bay, Kenya. PLoS One 7(3): e32140. 22427820 10.1371/journal.pone.0032140 PMC3299652

[28] World Health Organisation (1998) Laboratory service in tuberculosis control: Microscopy part II. Geneva: WHO press. 63p. Available: http://whqlibdoc.who.int/hq/1998/WHO_TB_98.258_(part2).pdf. Accessed 7 June 2011.

[29] Landis JR, Koch GG (1977) The measurement of observer agreement for categorical data. Biometrics 33: 159–174. 843571

[30] Soto A, Solari L, Gotuzzo E, Acinelli R, Vargas D, et al (2011) Performance of an algorithm based on WHO recommendations for the diagnosis of smear-negative pulmonary tuberculosis in patients without HIV infection. Trop Med Int Health 16: 424–30. 21208352 10.1111/j.1365-3156.2010.02715.x

[31] Bah B, Massari V, Sow O, Siriwardana M, Camara LM, et al (2002) Useful clues to the presence of smear-negative pulmonary tuberculosis in a West African city. Int J Tuberc Lung Dis 6: 592–8. 12102298

[32] Tamhane A, Chheng P, Dobbs T, Mak S, Sar B, et al (2009) Predictors of smear-negative pulmonary tuberculosis in HIV-infected patients, Battambang, Cambodia. Int J Tuberc Lung Dis 13: 347–54. 19275795

[33] Hepple P, Novoa-Cain J, Cheruiyot C, Richter E, Ritmeijer K (2011) Implementation of liquid culture for tuberculosis diagnosis in a remote setting: lessons learned. Int J Tuberc Lung Dis 15 3: 405–7. 21333111

[34] Saranchuk P, Boulle A, Hilderbrand K, Coetzee D, Bedelu M, et al (2007) Evaluation of a diagnostic algorithm for smear-negative pulmonary tuberculosis in HIV-infected adults. S Afr Med J 97: 517–23. 17805454

[35] Siddiqi K, Walley J, Khan MA, Shah K, Safdar N (2006) Clinical guidelines to diagnose smear-negative pulmonary tuberculosis in Pakistan, a country with low-HIV prevalence. Trop Med Int Health 11: 323–31. 16553912 10.1111/j.1365-3156.2006.01559.x

[36] Wilkinson D, Newman W, Reid A, Squire SB, Sturm AW, et al (2000) Trial-of-antibiotic algorithm for the diagnosis of tuberculosis in a district hospital in a developing country with high HIV prevalence. Int J Tuberc Lung Dis 4: 513–8. 10864181

[37] Kudjawu Y, Massari V, Sow O, Bah B, Larouzé B, et al (2006) Benefit of amoxicillin in differentiating between TB suspects whose initial AFB sputum smears are negative. Int J Tuberc Lung Dis 10: 441–6. 16602410

[38] World Health Organization (2010) Report on conclusions and recommendations. Strategic and Technical Advisory Group For Tuberculosis. Geneva: WHO press. Available: http://www.who.int/tb/advisory_bodies/stag_tb_report_2010.pdf. Accessed 31 August 2011.

