# Supplementary material for: Performance of the 2007 WHO Algorithm to Diagnose Smear-Negative Pulmonary Tuberculosis in a HIV Prevalent Setting
Source: PLoS One. 2012 Dec 19;7(12):e51336. doi: 10.1371/journal.pone.0051336 (PMC3526594; doi:10.1371/journal.pone.0051336)
Supplement: Figure S1 — Form used for X-ray interpretation in the prospective study. (PDF) [file pone.0051336.s001.pdf]

CXR FINDING

Date of CXR: |__||__| /|__|__| /|__|__|__|__|
Day Month Year

Quality of radiograph				
**Good quality** (No faults)		*Not good but usable, reasons*		
**Not good but usable** (some faults)		- **Too dark**	Cannot see peripheral vessels	
**Bad quality – not usable***		- **Too light**	Cannot see vertebral ends behind the heart	
		- **Rotated**	Medial clavicles different distances from spinous process	
		- **Under-inspired**	Less than 6 anterior ribs at diaphragm mid clavicular line	
		- **Parts cut off**	e.g. costophrenic angles	
		- **Artefacts seen**	Dots, lines, metal, clothing that is not anatomy or disease	
		- **Movement blur**	Like a moving photo – lose sharp edges of heart	

Fill in a new CXR form

Diagram example	Record Distribution Zones

Granuloma Oval lesion

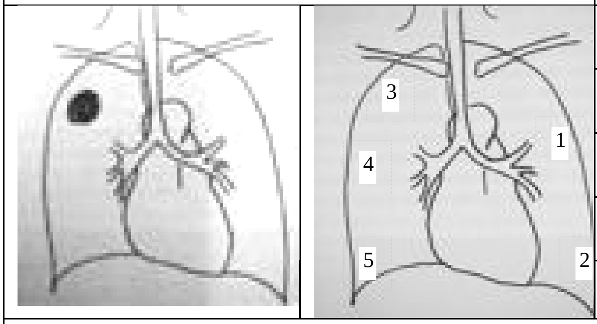

1	
2	
3	
4	
5	

Cavitation (within a granuloma,consolidation or infiltrate)

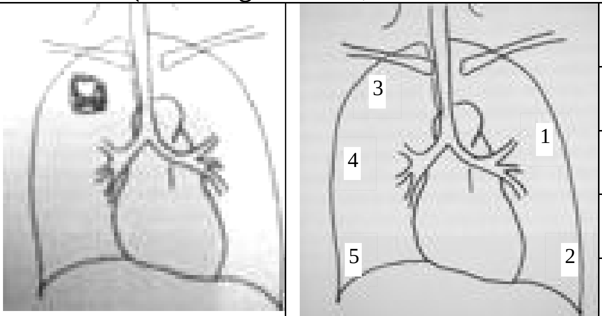

1	
2	
3	
4	
5	

Bronchopneumonic pattern, infiltration

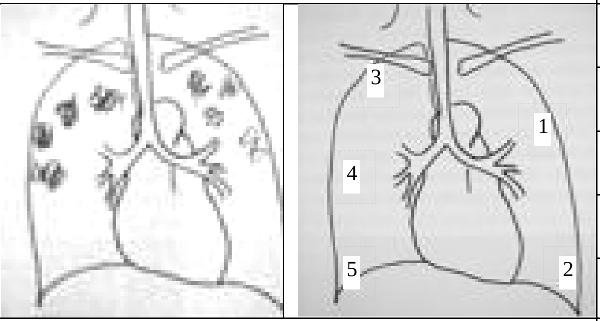

1	
2	
3	
4	
5	

Consolidation

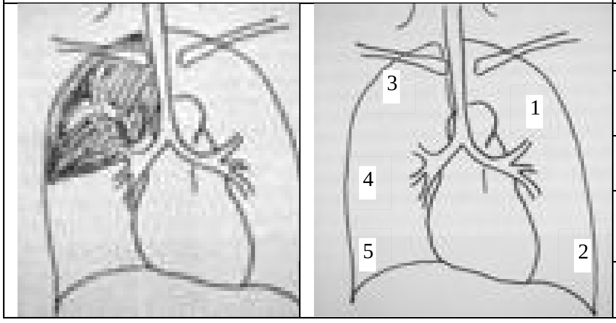

1	
2	
3	
4	
5	

Diagram example	Record Distribution Zones

Milliary Nodular pattern

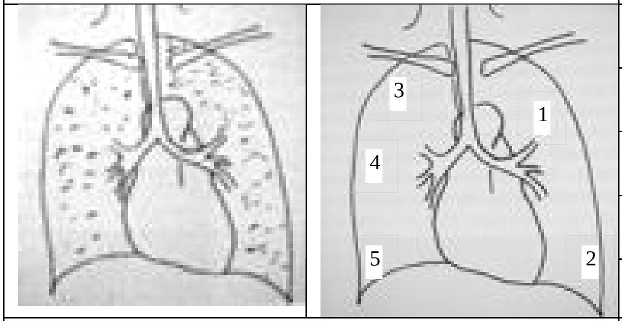

1	
2	
3	
4	
5	

Pleural effusion

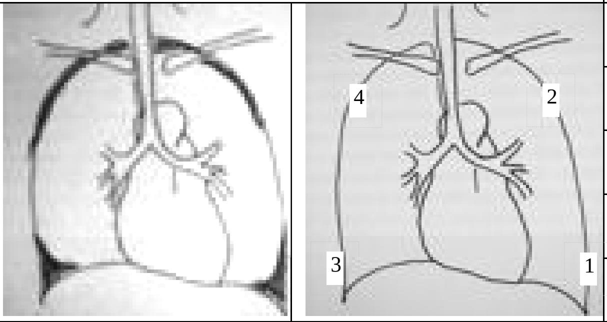

1	
2	
3	
4	

Lymph-adenopathy

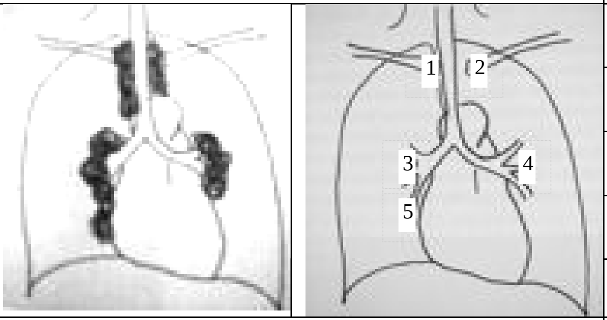

1	
2	
3	
4	
5	

Apical Fibrocystic Change Scarring and cysts

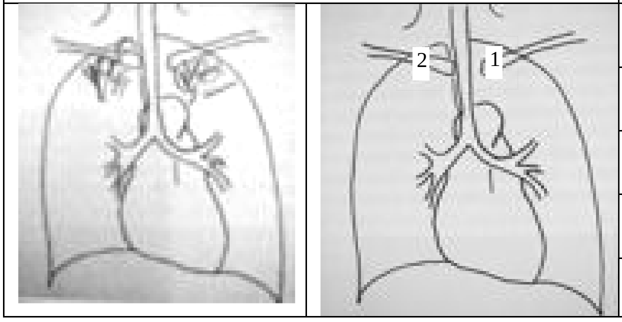

1	
2	

**Definitely TB**		**Maybe TB**		**Not TB**		
**Other diagnosis**						
